# Metastatic low-grade endometrial stromal sarcoma of uterus presenting as a primary pancreatic tumor: case presentation and literature review

**DOI:** 10.1186/s13000-019-0807-3

**Published:** 2019-04-22

**Authors:** Aoife J. McCarthy, Blaise A. Clarke, Ian McGilvray, Brendan C. Dickson, Korosh Khalili, Runjan Chetty

**Affiliations:** 1 Department of Anatomical Pathology, Laboratory Medicine Program, University Health Network, Toronto General Hospital, 200 Elizabeth Street, 11th Floor, Eaton Wing, Toronto, Ontario M5G 2C4 Canada; 2 Department of Laboratory Medicine and Pathobiology, Faculty of Medicine, University of Toronto, Toronto, Ontario Canada; 3 Surgery and Critical Care Program, Division of General Surgery, University Health Network, Toronto, Ontario Canada; 4 Department of Surgery, University of Toronto, Toronto, Ontario Canada; 5 Department of Laboratory Medicine, Mount Sinai Hospital, Toronto, Ontario Canada; 6 Joint Department of Medical Imaging, University of Toronto, Toronto, Ontario Canada; 7 Department of Radiology, University of Toronto, Toronto, Ontario Canada

**Keywords:** Endometrial stromal sarcoma, Extra-uterine, Low-grade, Pancreas

## Abstract

**Background:**

Metastatic tumors to the pancreas are uncommon, accounting for approximately 2% of pancreatic malignancies. The most common primary tumors to give rise to pancreatic metastases are carcinomas.

**Case presentation:**

A 50-year old female patient was investigated for a cause of abdominal discomfort. She had a 2-year history of menorrhagia and dysmenorrhea which was ascribed to a fibroid uterus. On imaging, she was found to have a large solid and cystic mass in the tail of the pancreas. Imaging also confirmed a fibroid uterus. A distal pancreatectomy and splenectomy showed a 9 cm circumscribed mass within, and grossly confined to, the parenchyma of the pancreatic tail.

Microscopically, the pancreatic lesion was lobulated, and well-circumscribed, but focally infiltrative. It comprised sheets of uniform spindled to epithelioid cells with round to oval nuclei, coarse to vesicular chromatin, visible nucleoli, nuclear grooves and clear to eosinophilic cytoplasm. Prominent arterioles were identified. The stroma was collagenized in areas. Occasional hemosiderin-laden macrophages were seen, and focal cystic change was present. There was no evidence of nuclear pleomorphism, mitotic activity or necrosis, and there was no evidence of endometriosis despite multiple sections being taken. Immunohistochemistry showed that the tumor cells were positive for CD10, estrogen receptor (ER), progesterone receptor (PR), Wilms tumor-1 (WT-1) and smooth muscle actin (SMA). RNA sequencing detected a *PHF1* rearrangement.

The morphological, immunohistochemical and molecular features were of a low-grade endometrial stromal sarcoma (LG-ESS). Subsequent total hysterectomy and bilateral salpingo-oophorectomy 3 months later, showed uterine fibroids and a 5 cm low-grade endometrial stromal sarcoma confined to the uterus, with lymphatic invasion.

**Conclusions:**

To the best of our knowledge, this is the first documented case of metastatic endometrial stromal sarcoma of uterus presenting as a primary pancreatic neoplasm. An unexpected extra-uterine location and unusual presentation of ESS may make the diagnosis challenging, despite classic histological features. Morphological, immunohistochemical and molecular findings must be combined to render the correct diagnosis.

## Background

Metastatic lesions to the pancreas are uncommon, and account for approximately 2% of pancreatic malignancies. The most common primary tumors to give rise to pancreatic metastases are carcinomas (e.g. from lung, gastrointestinal tract, kidney, breast); lymphoma, malignant melanoma and rarely sarcoma can also secondarily involve the pancreas [1, 2].

Low-grade endometrial stromal sarcoma (LG-ESS) is a rare uterine mesenchymal tumor, accounting for 0.2% of malignancies of the gynecologic tract. When uterine in location, endometrial stromal sarcoma (ESS) is readily diagnosed in view of the characteristic histology and patterns of invasion [3].

Primary, extra-uterine, endometrial stromal sarcomas (EU-ESS) also occur, but are very rare tumors that arise de novo at extra-uterine sites in the context of endometriosis, without uterine involvement [4]. The vast majority of documented cases have been low-grade, with only rare cases of high grade/dedifferentiated EU-ESS. In order to distinguish a primary EU-ESS from a metastasis from a primary uterine ESS, the absence of a primary uterine tumor must be confirmed by examination of a hysterectomy specimen or by imaging studies (MRI/PET-CT scan).

In the absence of a known primary uterine LG-ESS, the diagnosis of LG-ESS in an extra-uterine location, whether it represents metastatic disease from an as yet unknown uterine primary or primary EU-ESS, can be extremely challenging, as highlighted by our case report.

## Case report

We report the case of a 50-year old female patient, who was investigated for abdominal discomfort. She also had a 2-year history of menorrhagia and dysmenorrhea.

**Fig. 1:**
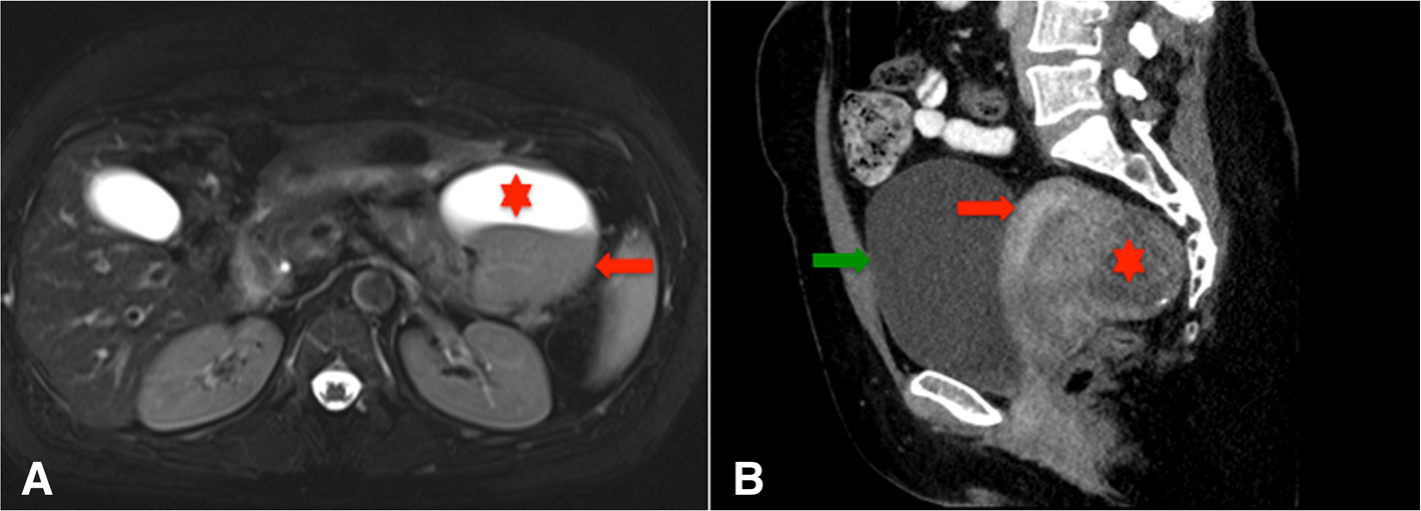
**a** (T2 axial image, MRI abdomen/pelvis); An MRI of abdomen and pelvis disclosed an 8 cm solid and cystic mass in the tail of the pancreas, which had a solid component (red arrow), as well as a hemorrhagic component (red *). **b** (sagittal image, CT of pelvis); A CT of pelvis confirmed a fibroid uterus, with a solid appearance to the lesions (green arrow, bladder; red arrow, uterus; red *, the largest uterine mass)

An ultrasound of abdomen showed the presence of a mass in the left upper quadrant, in keeping with a pancreatic mass. A CT and MRI of abdomen and pelvis confirmed the presence of an 8 cm solid and cystic mass in the tail of the pancreas (Fig. 1a). CT examination confirmed a fibroid uterus, while ultrasound showed the uterus to measure 7.7 × 6.6 × 4.7 cm with a 3 cm partially calcified posterior subserosal fibroid and an adjacent 4.7 cm partially cystic lesion, also deemed to be a fibroid (Fig. 1b).

A distal pancreatectomy and splenectomy was performed. A 9 cm circumscribed mass with yellow to tan solid and cystic cut surface was present in the tail of the pancreas, and was grossly confined to the pancreatic parenchyma. The mass was extensively sampled.

**Fig. 2:**
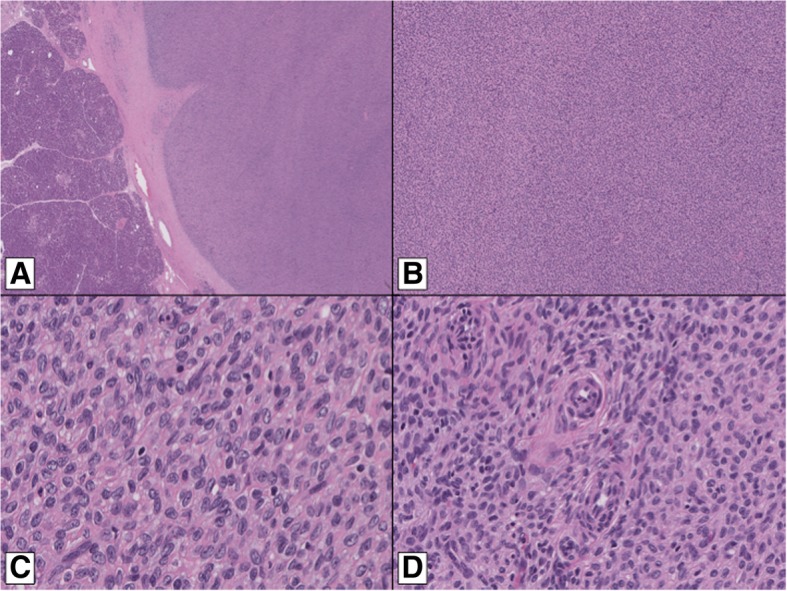
Histologically, the pancreatic lesion was lobulated, and predominantly well-circumscribed (**a**). It comprised sheets of uniform spindled to epithelioid cells (**b**). The lesional cells had round to oval nuclei, with coarse to vesicular chromatin, visible nucleoli, nuclear grooves and clear to eosinophilic cytoplasm (**c**). Prominent arterioles were seen (**d**)

Histologically, the lesion was lobulated, and predominantly well-circumscribed, but focally infiltrative (Fig. 2a), and was composed of sheets of uniform spindled to epithelioid cells (Fig. 2b). The lesional cells had round to oval nuclei, with coarse to vesicular chromatin, visible nucleoli, nuclear grooves and clear to eosinophilic cytoplasm (Fig. 2c). Prominent arterioles were identified (Fig. 2d). The stroma was collagenized in areas. Admixed lymphocytes, occasional hemosiderin-laden macrophages, and focal cystic change were present. There was no evidence of nuclear pleomorphism, mitotic activity or necrosis, and there was no evidence of endometriosis. Lymphovascular space invasion was not seen.

**Fig. 3:**
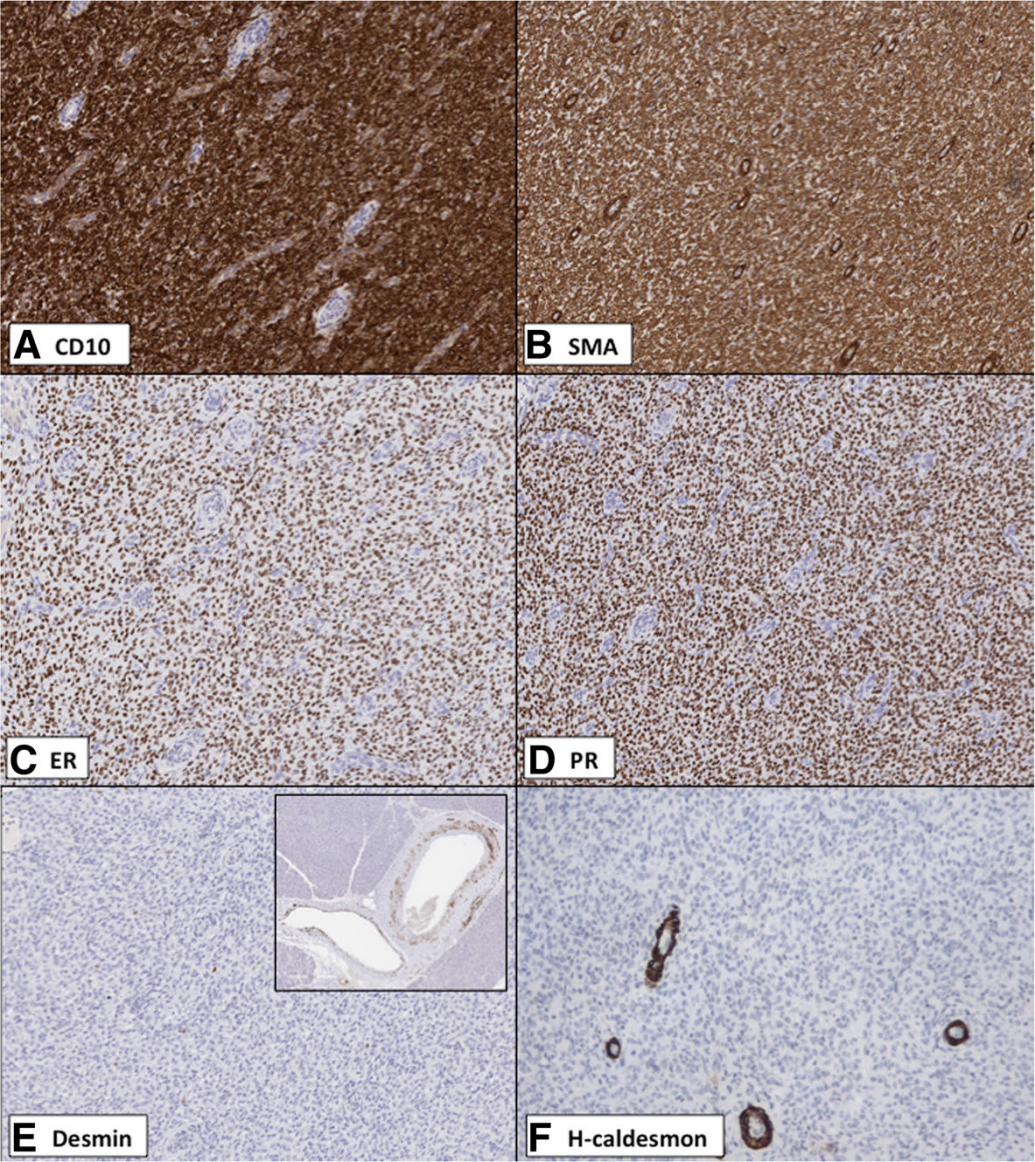
Morphologically, there was a high index of suspicion that the pancreatic lesion was an ESS, and this guided the selection of immunohistochemical markers. Immunohistochemistry showed that the tumor cells of the pancreatic lesion were positive for CD10 (**a**), smooth muscle actin (SMA) (**b**), estrogen receptor (ER) (**c**) and progesterone receptor (PR) (**d**). Desmin (**e**; inset of **e**: vessels in pancreatic parenchyma highlighted, acting as internal controls) and h-caldesmon (**e**; arterioles highlighted, acting as internal controls) were negative

Immunohistochemistry showed that the tumor cells were positive for CD10, estrogen receptor (ER), progesterone receptor (PR), Wilms tumor 1 (WT-1; nuclear staining) and smooth muscle actin (SMA) (Fig. 3). The tumor cells were negative for other smooth muscle markers (desmin, h-caldesmon) (Fig. 3), cytokeratins (AE1/3, CAM5.2), PAX-8, inhibin, and HMB-45.

RNA sequencing was performed using formalin-fixed paraffin-embedded tissue, cut into scrolls (4 cut at 10 μm). RNA was extracted using the ExpressArt FFPE Clear RNA Ready kit following manufacturer’s instructions (Amsbio, Cambridge, MA). The libraries were prepared using 20–100 ng of total RNA with the TruSight RNA Fusion Panel (Illumina, San Diego, CA). Each sample was sequenced with 76 base-pair paired-end reads using an Illumina MiSeq at eight samples per flow cell (~ 3 million reads per sample). The results were analyzed using both the STAR and BOWTIE2 aligners, and Manta and JAFFA fusion callers, respectively. Testing confirmed the presence of *PHF1* gene rearrangement (NM_024165.2).

The morphological, immunohistochemical and molecular features were of a low-grade endometrial stromal sarcoma (LG-ESS). Clinical and radiological correlation was required to determine if this lesion represented metastatic endometrial stromal sarcoma (ESS) from a uterine primary, or a rare primary extra-uterine endometrial stromal sarcoma (EU-ESS) of the pancreas, arising in the context of endometriosis. In view of the menorrhagia and dysmenorrhea, the patient underwent a total hysterectomy and bilateral salpingo-oophorectomy 3 months later. Histological examination of the uterus revealed fibroids and the presence of a 5.8 cm LG-ESS with evidence of lymphatic invasion. Thus, the ESS in the pancreas was clearly the result of a metastasis from a primary uterine ESS.

A CT of thorax, abdomen and pelvis performed 2 months post-hysterectomy showed no evidence of recurrent or additional metastatic disease. The case was reviewed by oncologists, and the decision was made to follow the patient with an MRI of abdomen and ultrasound of pelvis every 3 to 4 months for the foreseeable future, without adjuvant therapy.

## Discussion and conclusions

The natural history of LG-ESS is that of a slow-growing indolent tumor [5], but local relapses (i.e. pelvic, vaginal) [6] and/or distant metastasis (e.g. abdominal wall, lungs) [7, 8] do occur. LG-ESS rarely metastasizes to the pancreas.

In the case presented herein, a hysterectomy performed 6 months after the distal pancreatectomy, was confirmed to contain a LG-ESS, thereby confirming a uterine origin, with metastatic disease to the pancreas as the initial presentation of the tumor. Initial presentation of uterine LG-ESS as a pancreatic metastasis, as in our case, has not previously been described.

In extra-uterine sites, LG-ESSs display the typical morphological features of their uterine counterparts, consisting of sheets of monotonous, oval to fusiform cells, with scant cytoplasm, resembling stromal cells of normal proliferative-phase endometrium, interspersed with small vessels resembling spiral arterioles of the endometrium [9]. Typically, cytological atypia is minimal [3]. Nuclear grooves are often found diffusely throughout these lesions [10]. Mitotic activity is generally low (median mitotic index: 3 mitoses per 10 high power fields) [10]. In the uterus, ESS has a characteristic infiltrative, tongue-like pattern of invasion into the myometrium (finger-like projections extending > 3 mm from the border of the mass into surrounding tissue) and is frequently associated with lymphatic invasion, but this pattern of invasion may be lacking at extra-uterine sites, where the tumor typically has a multi-nodular appearance [10, 11].

Molecular characterization of uterine ESS has identified numerous genetic drivers. The t(7;17) translocation resulting in the *JAZF1*-*SUZ12* gene fusion is the most frequently reported genetic alteration found in uterine ESSs [9, 12–16]. Other chromosomal rearrangements reported in uterine ESSs include *PHF1* gene rearrangements resulting from t(6;10)(p21;p11) and t(6;7)(p21;p15), with fusion of *EPC1*-*PHF1* and *JAZF1*-*PHF1*, respectively [14, 17–19]. A minority of cases a minority involve other fusions, namely *MEAF6*-*PHF1*, *BRD8*-*PHF1*, *MBTD1*-*CXorf67*,11 and *EPC2*-*PHF1* fusions [20–23]. *EPC1*-*SUZ12* and *EPC1*-*BCOR* have recently been described in two ESSs; both tumors were characterized by an aggressive clinical course [24]. *JAZF1*-*SUZ12* fusions, *EPC1*-*PHF1* fusion, and *PHF1* rearrangement with no putative partner have been reported in extra-uterine low-grade endometrial stromal sarcomas [9, 12, 25–27]. In unusual sites, as in our case, molecular studies interpreted in the context of morphological and immunohistochemical features can aid in diagnosis.

The presence of a *PHF1* gene rearrangement, in a tumor with a multi-nodular growth pattern, comprising uniform round to oval tumor cells embedded in a fibro-myxoid stroma, outside the uterus, raises the possibility of ossifying fibromyxoid tumor (OFMT) [28]. Unlike ESS, however, OFMT generally contains spicules of metaplastic bone at the periphery of the nodules, and the tumor cells are often positive for S100 protein [28, 29]. Furthermore, *PHF1* gene rearrangements in OFMT are characterized by fusion to *EP400* in the majority of cases, and fusion to *MEAF6* and *EPC1* in a minority of cases [28, 30, 31], whereas in ESS, *PHF1* rearrangements are typically fused with either *JAZF1* or *EPC1* [19].

Once a diagnosis of ESS has been rendered in an extra-uterine location, it is essential to determine if one is dealing with a metastasis from a primary uterine ESS or a primary extra-uterine ESS [10]. When the uterus is removed along with the extra-uterine tumor, this distinction is possible as careful gross and microscopic evaluation of the endometrium and myometrium should exclude the presence of a primary tumor. When hysterectomy precedes removal of the extra-uterine tumor, or if the uterus is left in situ, then a definitive diagnosis relies on the review of prior surgical pathology material and of imaging studies, and thorough sampling of the extra-uterine tumor to assess for histological features that could aid in the exclusion of a uterine primary, namely endometriosis [10].

LG-ESSs rarely occur at extra-uterine sites (either as primary or secondary lesions), and when they do, pose challenges in diagnosis due to unexpected location, non-gynecological signs and symptoms, the presence of variant histological patterns, and the lack of a unique immunohistochemical profile. Awareness of presentation at extra-uterine locations and attention to histological features, in conjunction with immunohistochemical and molecular findings, are essential for making an accurate diagnosis.

## Abbreviations

ER: Estrogen receptor
ESS: Endometrial stromal sarcoma
EU-ESS: Extra-uterine, endometrial stromal sarcoma
LG-ESS: Low-grade endometrial stromal sarcoma
OFMT: Ossifying fibromyxoid tumor
PR: Progesterone receptor
SMA: Smooth muscle actin
WT-1: Wilms tumor-1
